# Integrating Hospice and Palliative Medicine Education Within the American Board of Emergency Medicine Model

**DOI:** 10.5811/westjem.18448

**Published:** 2024-03-25

**Authors:** Rebecca Goett, Jason Lyou, Lauren R. Willoughby, Daniel W. Markwalter, Diane L. Gorgas, Lauren T. Southerland

**Affiliations:** * Rutgers New Jersey Medical School, Department of Emergency Medicine, Newark, New Jersey; † The Ohio State University Wexner Medical Center, Department of Emergency Medicine, Columbus, Ohio; ‡ University of North Carolina School of Medicine, Department of Emergency Medicine, Chapel Hill, North Carolina; § University of North Carolina School of Medicine, UNC Palliative Care Program, Chapel Hill, North Carolina

## Abstract

**Background:**

Hospice and palliative medicine (HPM) is a board-certified subspecialty within emergency medicine (EM), but prior studies have shown that EM residents do not receive sufficient training in HPM. Experts in HPM-EM created a consensus list of competencies for HPM training in EM residency. We evaluated how the HPM competencies integrate within the American Board of Emergency Medicine Milestones, which include the Model of the Clinical Practice of Emergency Medicine (EM Model) and the knowledge, skills, and abilities (KSA) list.

**Methods:**

Three emergency physicians independently mapped the HPM-EM competencies onto the 2019 EM Model items and the 2021 KSAs. Discrepancies were resolved by a fourth independent reviewer, and the final mapping was reviewed by all team members.

**Results:**

The EM Model included 78% (18/23) of the HPM competencies as a direct match, and we identified recommended areas for incorporating the other five. The KSAs included 43% (10/23). Most HPM competencies included in the KSAs mapped onto at least one level B (minimal necessary for competency) KSA. Three HPM competencies were not clearly included in the EM Model or in the KSAs (treating end-of-life symptoms, caring for the imminently dying, and caring for patients under hospice care).

**Conclusion:**

The majority of HPM-EM competencies are included in the current EM Model and KSAs and correspond to knowledge needed to be competent in EM. Programs relying on the EM Milestones to plan their curriculums may miss training in symptom management and care for patients at the end of life or who are on hospice.

## INTRODUCTION

A third of adults who die will receive emergency department care in the month prior to their death.^1^ Emergency physicians need training to provide the high-quality, goal-concordant care that these patients deserve. Hospice and palliative medicine (HPM) is a subspecialty of emergency medicine (EM) that adds an additional focus on symptom management, goal-concordant care, and quality of life, especially for patients with chronic disease or life-threatening conditions, or who are at the end of life.^2^ Prior research has shown that current EM residency training lacks instruction in HPM.^3^ ^–^ ^7^ To address this, the American College of Emergency Physicians Palliative Medicine Section published a list of 23 critical developmental milestones in HPM training for EM residents.^8^ However, it is unclear how best to integrate these recommendations into an EM residency curriculum.

Many EM residency curriculums are based on the knowledge needed to pass the EM board certification exams. This knowledge is codified in the American Board of Emergency Medicine (ABEM) Model of the Clinical Practice of Emergency Medicine (EM Model) and a list of knowledge, skills, and abilities (KSA).^9^ ^,^ ^10^ The EM model along with the KSAs are the foundational documents used to create the EM Milestones, a compendium ubiquitously employed in both EM training and assessment. Our goal in this study was to determine where the HPM competencies fit or could fit within the EM Model and KSAs. This mapping could help guide curriculum design or the incorporation of the HPM competencies into testing content.

## METHODS

This study was not human subjects research and was deemed exempt from institutional review board review. We compared the 2019 EM Model and the 2021 KSAs to the HPM competencies. The HPM competencies were assigned numerals. The EM Model items were annotated by their number and category. The notations for the KSA categories and codes were used directly from the 2021 document. We divided the KSAs into overarching categories (eg, diagnosis, pharmacotherapy, reassessment) which we then further divided into sets of competencies within that category.^10^ Each competency was given a hierarchy in training corresponding to an alphabetic level (with A the most advanced level of competency and E the least). Level A is reserved for advanced knowledge or skills. Level B is the minimal competency level, defined as the minimum skill level every EM resident should attain to graduate. Levels C, D, and E are skills in the development of reaching level B.

As this type of analysis has not been done before, we used a sequential approach with initial independent reviewers, a mediator step, and then final consensus group discussion. The consensus group results were then reviewed by two independent external experts. In the first phase of consensus mapping, two residents (EM postgraduate year (PGY)-2 and EM/internal medicine (PGY-4) and an EM attending independently mapped palliative care competencies using a Microsoft Excel spreadsheet (Microsoft Corporation, Armonk, NY). The three initial concept mappers had independent data sheets and were blinded to each other’s determinations. A competency could map onto more than one area of the EM Model. First, keywords from each HPM competency were searched for in the EM Model. If no matches were found, the EM Model was reviewed line by line to determine whether there were conceptual matches. If there was no direct match, but the HPM competency could be incorporated under a topic, this was listed as a potential area for incorporation.

Any topic that did not have at least 2/3 agreement on the initial independent review was reviewed by a fourth emergency physician with expertise in EM resident education and EM Model development. She was blinded to the initial reviewer’s names but did have their results. The full group met and reviewed all the mapping until consensus was reached. The consensus tables were then reviewed independently by two additional external HPM board-certified EM attendings involved in resident education at two different EM residency programs. The same process was used for mapping the KSAs.

## RESULTS

### Incorporation into the Emergency Medicine Model

Fifty-one of 963 EM Model items were tagged in the independent first round of mapping, with 98.7% consensus (951/963) between the initial three independent reviewers on whether an item was or was not tagged as a match. The final review by the independent HPM-boarded EM attendings did not result in adjustments to any of the existing mapping but did add to the potential areas of fit for the HPM competencies that did not directly match onto the EM Model. Table 1 lists the competencies included in the 2019 EM Model (18/23, 78%). Many competencies fit into *EM Model category 20: Other Core Competencies* section, which includes communication skills, transitions of care, cultural competency, and healthcare coordination. Discrepancy discussions centered around management vs diagnosis. The competency *HPM 2: Treating distressing symptoms (eg, nausea/vomiting, dyspnea)* was felt to fit by keyword match under EM Model category *1.0 Signs, Symptoms and Presentations*. However, that category does not mention treatment of symptoms directly. Similarly, *HPM 18: Complications of Cancer* could map to many items in the EM model, but again refers to palliative management of cancer complications rather than diagnosis.

**Table 1.** The hospice and palliative emergency medicine residency education competencies mapped onto the American Board of Emergency Medicine EM Model.

Hospice and palliative competency	Description	EM model item
1	Pain control: a. chronic pain, b. malignant and non-malignant pain.	19.3.1 Anesthesia and acute pain management- regional anesthesia
		19.3.2 Anesthesia and acute pain management- procedural sedation
		19.3.3 Anesthesia and acute pain management- analgesia
2	Treating distressing symptoms (eg, nausea/vomiting, dyspnea)	1.3.32 Nausea/vomiting 1.3.42 Shortness of breath *unclear whether these EM model elements refer to treating these symptoms or developing a differential diagnosis for these symptoms, but both should be taught.
3	Difficult communication: a. delivery of bad news (eg, prognosis and death telling) b. conflict resolution (eg, between family members	20.1.2.2 Interpersonal and communication skills- conflict management
		20.1.2.4 Interpersonal and communication skills- delivering bad news/death notifications
4	Goals of care discussions: a. assisting families with decision making. b. assisting patients with decision making	20.4.4.1 Health care coordination- advance directives
5	Caregiver support	20.3.4.6 Well-being and resilience- care for the caregiver
6	Non-initiation or stopping of nonbeneficial interventions	19.2 Resuscitation- cardiopulmonary resuscitation
		20.1.1.3 Interpersonal skills- patient and family experience of care
		20.4.4.2.2 Healthcare coordination- withdrawal of support
9	Bereavement and grieving	14.2.4 Mood disorders and thought disorders- grief reaction
10	Family-witnessed resuscitation	19.2 Resuscitation- cardiopulmonary resuscitation
12	Coping and self-care	20.3.4.1 Well-being and resilience- fatigue and impairment
		20.3.4.1.1 Well-being and resilience- sleep hygiene
		20.3.4.3 Well-being and resilience- work/life balance
13	End-of-life management in the mass casualty incident/event	20.4.2.2.1 Patient triage and classification
16	Screening for palliative care needs: a. identifying patients who may benefit from HPM specialist referral, b. identifying the imminently dying patient (expected death within hours-days).	20.4.4.2.1 Health care coordination- patient identification for palliative care
		20.4.4.2.3 Health care coordination- hospice referral
17	Rapid palliative care assessment: a. aligning diagnostics and therapeutics to patient goals, b. functional, psychosocial, and spiritual assessment, c. assessing for and initiating hospice referrals, d. toolkits to help identify patient needs for appropriate referrals/resources, e. caregiver burden.	20.3.4.6 Well-being and resilience- care for the caregiver
		20.4.4.2.3 Healthcare coordination- hospice referral
		20.4.4.3.1 Healthcare coordination- activities of daily living/functional assessment
18	Complications of cancer: a. disease complications (eg, spinal cord compression, hypercalcemia), b. treatment complications (eg, pancreatitis, tumor lysis, neutropenia, acute renal failure).	2.9.2.3 Large bowel- radiation colitis
		2.9.2.5 Large bowel- neutropenic enterocolitis/typhlitis
		3.6.1 Diseases of the pericardium- pericardial tamponade
		8.7 Oncologic emergencies
		8.7.1 Oncologic emergencies- febrile neutropenia
		8.7.2 Oncologic emergencies- hypercalcemia of malignancy
		8.7.3 Oncologic emergencies- hyperviscosity syndrome
		8.7.4 Oncologic emergencies- malignant pericardial effusion
		8.7.5 Oncologic emergencies- spinal cord compression
		8.7.6 Oncologic emergencies- superior vena cava syndrome
		8.7.7 Oncologic emergencies- tumor hemorrhage
		8.7.8 Oncologic emergencies- tumor lysis syndrome
		11.1.4.2 Bony abnormalities-tumor-related fractures
		16.2.3 Disorders of the pleura, mediastinum, and chest wall-pleural effusion
		16.6.2 Pulmonary embolism/infarct- venous thromboembolism
		16.6.2.1 Pulmonary embolism/infarct- massive and submassive embolism
19	Ethical, spiritual, and cultural issues around end-of-life and death	20.1.2.5 Interpersonal and communication skills- cultural competency
20	Advance directives: a. physician order for life-sustaining treatment (POLST), b. medical order for life-sustaining treatment (MOLST), c. five wishes.	20.4.4.1 Healthcare coordination- advance directives
21	ethical and legal issues: a. decision-making capacity, b. futility.	20.3.2.4 Professionalism- medical ethics
		20.4.5.4 Regulatory/legal- consent, capacity and refusal of care- consent, capacity and refusal of care
22	Multidisciplinary team and support systems. (understanding team roles and system resources): a. spiritual chair, b. social chair, c. hospice care eligibility, d. continuing care, e. importance of local and community support systems.	20.1.1.1 Interpersonal skills- inter-departmental and medical staff relations
		20.1.1.2 Interpersonal skills- intra-departmental relations, teamwork, and collaboration skills
		20.4.2.4.1 ED administration- allied health professionals
23	Transitions across care settings, eg, inpatient vs home hospice, palliative care unit	20.4.4.2.1 Healthcare coordination- patient identification for palliative care
		20.4.4.2.3 Healthcare coordination- hospice referral

### Potential Areas of Fit in the Emergency Medicine Model

Five HPM competencies did not fit into the EM Model. The first two, *HPM 7: Treating common end-of-life symptoms* and *HPM 8: Care for the imminently dying (expecting death within hours to days or recently deceased patient and their family members)*, could be taught under EM Model item *20.4.4.2.2: Systems-based Practice: Withdrawal of support.* This EM Model item could be clarified to ensure that it includes symptom control and end-of-life care. The next, *HPM 11: Caring for patients under hospice care,* could be taught when teaching *20.4.4.2.3: Systems-based Practice: Hospice Referral.* However, the hospice-referral EM Model item better mapped onto *HPM 17*, which includes assessing for and initiating hospice referrals. The team felt that identifying and referring patients to hospice was a separate skillset than caring for patients on hospice. The last two HPM competencies without a clear association with the EM Model were *HPM 14: Trajectories of dying: a. Terminal illness, b. Organ Failure, c. Frailty, d. Sudden Death,* and *HPM 15: Prognostication.* While these competencies necessitate having sound understanding of the natural history of disease as well as physical examination and clinical workup components informing prognosis, these are also skills for explaining the likelihood of death and communicating with patients and families. The team consensus was that these could be taught within the EM Model items *20.1.2.4 Interpersonal and Communication Skills: Delivering bad news/Death Notifications* and *20.1.1.3 Interpersonal and Communication Skills: Patient and family experience of care.*

### Incorporation into the Knowledge, Skills and Abilities

Thirty items of 214 were tagged in the first round with 87% consensus (187/214) between the initial three independent reviewers on whether an item was or was not tagged as a match. Ten of the 23 HPM competencies (43%) mapped onto 16 different KSAs (Table 2). Of the 16 matches within the KSAs, none were advanced skills (level A). All but HPM 13 mapped onto at least one level B skill. A table showing all the HPM competencies and their incorporation within the EM Model and KSAs together is included as Supplemental Data A.

**Table 2.** The palliative emergency medicine competencies incorporate with the 2021 American Board of Emergency Medicine knowledge, skills, and abilities.

	Hospice and palliative medicine competency	KSA code	Description	Level
3	Difficult communication a. delivery of bad news (eg, prognosis and death telling) b. conflict resolution (eg, between family members)	CS17	Use flexible communication strategies to negotiate effectively with staff, consultants, patients, families, and others to provide optimal patient care, recognizing and resolving interpersonal conflicts	B
4	Goals of care discussions: a. assisting families with decision making. b. assisting patients with decision making.	CS3	Elicit patients’ reasons for seeking healthcare and their expectations from the ED visit	D
		CS7	Consider the expectations of those who provide or receive care in the ED and use communication methods that minimize the potential for stress, conflict, and miscommunication	B
		CS15	Solicit patient participation in medical decision-making by discussing, risks, benefits, and alternatives to care provided	C
		ES15	Elicit the patient’s goals of care prior to initiating emergency stabilization, including evaluating the validity of advanced directives	B
13	End-of-life management in the mass casualty incident/event	DM11	Participate in a mass casualty drill or event in an ED involving multiple patients, prioritizing care, containing potential exposures, and appropriately assigning resources	C
14	Trajectories of dying: a. terminal illness, b. organ failure, c. frailty, d. sudden death.	ES6	Recognize in a timely fashion when further clinical intervention is futile	B
		PE6	Educate patients on the natural course of their disease and impact of possible treatment in relation to prognosis	B
15	Prognostication	ES6	Recognize in a timely fashion when further clinical intervention is futile	B
		ES15	Elicit the patient’s goals of care prior to initiating emergency stabilization, including evaluating the validity of advanced directives	B
		PE6	Educate patients on the natural course of their disease and impact of possible treatment in relation to prognosis	B
		TC11	Determine, summarize, and communicate the diagnosis or diagnostic uncertainty, anticipated course, prognosis, disposition plan, medications, future diagnostic/therapeutic interventions, signs and symptoms for which to seek further care and follow-up to patient or surrogate	B
17	Rapid palliative care assessment: a. aligning diagnostics and therapeutics to patient goals, b. functional, psychosocial, and spiritual assessment, c. assessing for and initiating hospice referrals, d. toolkits to help identify patient needs for appropriate referrals/resources, e. caregiver burden.	CS7	Consider the expectations of those who provide or receive care in the ED and use communication methods that minimize the potential for stress, conflict, and miscommunication	B
20	Advance directives: a. physician order for life-sustaining treatment (POLST), b. medical order for life-sustaining treatment (MOLST), c. five wishes.	CS6	Elicit information from patients, families, and other healthcare members using verbal, nonverbal, written, and technological skills	D
		ES15	Elicit the patient’s goals of care prior to initiating emergency stabilization, including evaluating the validity of advanced directives	B
21	Ethical and legal issues: a. decision-making capacity, b. futility.	CS15	Solicit patient participation in medical decision-making by discussing, risks, benefits, and alternatives to care provided	C
		ES6	Recognize in a timely fashion when further clinical intervention is futile	B
		LI12	Balance patient autonomy with patient protection and advocacy when addressing consent and refusal of care in accordance with legal and ethical standards	B
		TI9	Obtain informed consent from the patient or appropriate surrogate when indicated	B
22	Multidisciplinary team and support systems. (understanding team roles and system resources): a. spiritual chair, b. social chair, c. hospice care eligibility, d. continuing care, e. importance of local and community support systems.	TM1	Organize patient care teams	B
23	Transitions across care settings, eg, inpatient vs home hospice, palliative care unit	CS5	Communicate information to patients and families using verbal, nonverbal, written, and technological skills, and confirm understanding	B
		CS10	Communicate pertinent information to healthcare colleagues in effective and safe transitions of care	C
		TC11	Determine, summarize, and communicate the diagnosis or diagnostic uncertainty, anticipated course, prognosis, disposition plan, medications, future diagnostic/therapeutic interventions, signs and symptoms for which to seek further care and follow-up to patient or surrogate	B
		TC15	Ensure transitions of care are accurately and efficiently communicated between clinicians using best practices	B

### Potential Areas of Fit into the Knowledge, Skills and Abilities

Three additional KSAs were identified as having areas of potential fit or incorporation. *HPM 5: Caregiver support* and *HPM 12: Coping and self-care* could be taught while discussing *CS2: Establish rapport with and demonstrate empathy toward patients and their families*. Finally, *HPM 16: Screening for palliative care needs* could be taught with *TC18: Correctly determine the appropriate disposition*.

## DISCUSSION

This study showed fair to good inclusion of HPM competencies within the published EM KSAs and EM Model, demonstrating that the HPM competencies are represented in the Milestones. However, key topic areas were identified that could improve the focus of EM training in HPM. Demonstrating the overlap of the HPM and EM content may help EM educators ensure that HPM training is incorporated into their curriculums. Lack of training on these topics is a consistent finding in national and international studies, and educators need better ways to incorporate HPM-EM training into residency curriculums.^3^ ^–^ ^7^ ^,^ ^11^ ^–^ ^13^ Improved teaching of the HPM-EM competencies has the potential to decrease the care gaps seen in ED symptom management and end-of-life care, including lack of goals of care conversations for critically ill patients.^14^ ^,^ ^15^

A limitation of the HPM competencies is that they have not been externally assessed or investigated and are based on expert consensus. None of the initial four reviewers were involved in the development of the HPM competencies and they found them to almost all map onto the EM Model or identified places in the EM Model that could be expanded to include them more explicitly. Additionally, the HPM competencies that mapped onto KSAs all met at least one KSA on the minimal competency level. These findings imply that the HPM competencies are skills that are at resident level.

The descriptions in the HPM competencies can add depth to the corresponding EM Milestones for curriculum development and summative evaluation. For example, most residencies provide training or simulations of mass casualty care. The study group envisioned ways in which end-of-life management could be added into that training (HPM 13). Likewise, a lecture on post-cardiac arrest care could incorporate training on the non-initiation or compassionate discontinuation of interventions such as mechanical ventilation (HPM 6). Summative competency assessments at end of training to gain board certification could also incorporate more HPM competency-based questions.

Much of the overlap between the HPM competencies and the EM Model and KSAs was in *Interpersonal and Communication Skills* (EM Model) and the *CS – Communication & Interpersonal Skills* (KSAs). Communication skills, although challenging to teach, are critical in patient-centered care and will likely have an increased emphasis as artificial intelligence and machine learning become more universally integrated into clinical care. Current models for communication instruction rely heavily on role modeling.^16^ Residents have suggested that formal training in communication should focus on general communication skills and should provide syntax to use in future discussions.^17^ Developing communication skills requires deliberate practice of techniques, including NURSE statements (naming, understanding, respecting, supporting, and exploring) and Ask-Tell-Ask.^17^ ^,^ ^18^ Additionally, educators must become familiar with methods for real-time teaching of communication, such as “Could I add something?”^19^

Trajectories of dying (HPM 7) and prognostication (HPM 8) are two skills used to counsel patients/families with serious illness or at the end of life that did not fit clearly within the EM Model. These are difficult skills, and prior studies have identified some discordance between what families/caregivers understand about a person’s death and the underlying causes of death identified by the physician-led team.^20^ Thus, this skill should be honed throughout training. It is our experience that EM residents rarely receive explicit education on prognostication, and so we recommend its incorporation into curriculums. Our results further suggest that training on treating end-of-life symptoms, care for the imminently dying, and caring for patients under hospice care could be overlooked by current resident curriculums with strict adherence to the EM Model.

## LIMITATIONS

A limitation of this project is that even though a consensus process was used with experts in residency education and HPM, other education experts may interpret the domains and competencies differently. For example, the *EM Model item 20.3.4.6 Well-being and Resilience - Care for the caregiver* was matched to HPM 5 and 17 about patient caregivers. However, this could also be interpreted as resident self-care as it is under the well-being section. Finally, while trained HPM emergency physicians reviewed all the mapping, the initial mapping did include resident input. This could be considered an advantage, as they are experiencing lectures weekly, or are a potential source of bias, as they have not had a full EM curriculum yet.

## CONCLUSION

We identified areas of overlap where the HPM-EM subspecialty competencies can be emphasized or integrated into EM Model-based residency curriculums. This knowledge can be used for curriculum planning and incorporating HPM into definitions for competency in EM. These could also be reflected in final summative evaluations for certification.
